# Incidence and Determinants of Nevirapine and Efavirenz-Related Skin Rashes in West Africans: Nevirapine's Epitaph?

**DOI:** 10.1371/journal.pone.0094854

**Published:** 2014-04-11

**Authors:** Fred Stephen Sarfo, Maame Anima Sarfo, Betty Norman, Richard Phillips, David Chadwick

**Affiliations:** 1 Department of Medicine, Komfo Anokye Teaching Hospital, Kumasi, Ghana; 2 Department of Medicine, Kwame Nkrumah University of Science and Technology, Kumasi, Ghana; 3 Centre for Clinical Infection, The James Cook University Hospital, Middlesbrough, United Kingdom; University of Buea, Cameroon

## Abstract

Non-nucleoside reverse transcriptase inhibitor (NNRTI) associated rash is common and frequently leads to discontinuation of NNRTIs. This study assessed the risk of developing rashes and discontinuing NNRTIs and associated factors in a large clinic in central Ghana. In this retrospective cohort study, clinical data were obtained in patients starting efavirenz or nevirapine between 2004–2010. Factors associated with rashes were explored using a multivariate Cox proportional hazards regression model. Of 3,999 patients who started NNRTI-based ART, 281 (7.0%) experienced at least one episode of NNRTI-related rash with an incidence of 2.63 events/100 person-years, occurring in 10.2% and 5.6% of patients taking nevirapine and efavirenz respectively. Most rashes (94%) were grade 1 or 2 and were reported a median of 2 months following initiation of ART. In multivariate analysis developing a rash was associated with nevirapine use (aHR 1.67, 95% CI 1.28–2.10), female gender (aHR of 1.39, 95% CI 1.01–1.92) and lower baseline CD4 counts (aHR 0.88, 95% CI 0.82–0.95 per 50 cells/mm^3^ increment). Patients with nevirapine-associated rash were 11 times more likely to discontinue treatment as patients with efavirenz-associated rash. In contrast to findings in other studies, NNRTI-associated rashes in Ghanaians appear more common in patients with lower baseline CD4 counts. Given the increased frequency of rashes with nevirapine and subsequent discontinuations in many patients, along with other treatment-limiting toxicities, this provides further impetus for the replacement of nevirapine by efavirenz as the first-line NNRTI treatment of choice in Africa.

## Introduction

There has been a rapid and successful scaling-up of antiretroviral therapy (ART) in most resource-constrained countries over the past decade. As of December 2011 in sub-Saharan Africa there was a combined total of 6.2 million children and adults on ART out of an estimated 11 million people needing ART [1]. In line with World Health Organisation (WHO) recommendations at the start of the ART roll-out, most national programmes implemented first line treatment comprising a dual nucleoside reverse transcriptase inhibitor (NRTI) backbone of either of zidovudine (AZT) or stavudine (d4T) plus lamivudine (3TC), with a non-nucleoside reverse transcriptase inhibitor (NNRTI): either nevirapine or efavirenz [2]. More recently the WHO has recommended efavirenz as the preferred first line NNRTI in resource-limited settings [3]. The durability of these first line ART regimens is dependent on their efficacy and toxicity profile over the long-term. Adverse effects have been reported with all antiretroviral drugs and are one of the most common reasons for discontinuation of treatment [4], [5].

The NNRTIs are highly effective but are limited by the development of adverse events such as hypersensitivity, predominantly cutaneous and hepatotoxic reactions. NNRTI-related rashes occur rapidly, usually within the first few weeks or months of starting treatment and may be associated with severe morbidity and occasional mortality. The incidence and risk factors for these cutaneous perturbations has seldom been explored among cohorts from sub-Saharan Africa. In the 2NN trial, the incidence of NNRTI-related skin rash was high and was associated with use of nevirapine, female gender and less advanced HIV disease status in a sub-study among a Thai cohort [6]. In sub-Saharan Africa, WHO surveillance data have indicated that most treatment programmes use predominantly nevirapine rather than efavirenz, mainly due to cost [7]. In a recent study comparing the long-term effectiveness of NNRTI-based ART in Ghana, rashes were the most frequently-observed toxicity leading to treatment discontinuation [8]. Given that toxicity of ART may cause poor adherence to therapy and therefore engender treatment failure it is important to explore risk factors associated with the incidence of severe toxicity among the currently available first line therapies. We report on the incidence and determinants of NNRTI-related skin rashes in a large cohort of Ghanaian HIV-infected patients starting NNRTI-based ART.

## Methods

### Patients and data collection

Ethical permission for this study was provided by the Committee on Human Research Publications and Ethics of the Kwame Nkrumah University of Science and Technology and the Komfo Anokye Teaching Hospital (KATH), Kumasi, Ghana (ref: CHRPE/AP/073/13). Our institutional review board waived the need for a written informed consent since this was a retrospective, observational study and anonymised data were collected from patients' records. Since 2004, patients referred to the HIV clinic in KATH, Kumasi have been treated as part of the National AIDS Control Program. Patients were referred from a large area of central and northern Ghana and after starting ART reviewed after 2 months then every 6 months subsequently. HIV viral load was not available routinely and testing for HIV-2 and HBV co-infection has been performed only in limited circumstances. The criteria for starting ART in Ghana followed the WHO guidelines [2], with a change of the CD4 threshold for initiation from 200 to 350 cells/mm^3^ in 2008. First-line ART comprised lamivudine plus either zidovudine or stavudine, plus either nevirapine or efavirenz. The choice between the use of either zidovudine or stavudine was determined by availability but zidovudine was avoided in patients with haemoglobin below 10 g/dL. For NNRTIs, nevirapine tended to be used preferentially in women of child-bearing age. Data on patients' response to ART including adverse side effects are routinely sought for and documented in case notes at each clinic appointment.

### Skin rash definitions

The types and severity of skin rash were documented and described by clinicians in patients' folders. Based on these descriptive accounts severity of skin rash was staged, using criteria published in the 2NN sub-study [6] as Level I: erythema or hyper-pigmented rash; level IIA: diffuse maculopapular rash; level IIB: urticaria; level III: rash plus constitutional symptoms such as fever, myalgia, pruritus and malaise or angioedema, serum sickness-like reactions, Steven's Johnson syndrome; level IV: toxic epidermal necrolysis. A cross-reactive NNRTI-cutaneous reaction was defined as the re-appearance of a rash attributable to NNRTI, after substituting one NNRTI for another. When the physician did not attribute skin rash to drug toxicity, the cause of the skin rash was recorded but not included in the analysis for toxicity.

### Statistical analysis

Parametric and non-parametric methods were used to compare baseline characteristics of continuous data between patients with and without NNRTI-related skin rash. Mann-Whitney's U-test was used to compare the two groups since the baseline continuous data were independent between the patients. Comparisons of dichotomous data were performed using Chi-squared or Fisher's exact test. The cumulative incidence of NNRTI-related skin rash was calculated using Kaplan-Meier analysis. Patients were censored either at the date of first NNRTI-related skin rash, at the last clinic visit for patients that died, were transferred out or were lost to follow-up, and at December 31 2011 for the remainder. Patients who switched to second line ART regimens due to either clinical or immunological failure were censored at the date of switching. A risk factor analysis was performed using a multivariate Cox proportional hazards regression model. Collinearity between variables was assessed and a backward selection method was used, retaining those variables with p-values <0.05 in the final model. The level of significance was set at p<0.05. All analyses were performed using SPSS version 17.

## Results

### Baseline characteristics of patients

Prior to initiation of ART, patients who subsequently developed a NNRTI-related rash (compared with those who did not) were more likely to be females, had lower median CD4 counts, lower serum creatinine concentration, were more likely to be hepatitis B surface antigen (HBsAg) positive and initiate nevirapine (over efavirenz), as shown in Table 1. However no significant differences were present between the two groups with respect to their median age, WHO clinical stages, median body mass index (BMI), serum transaminases as well as the NRTI backbone used to initiate therapy. Patients starting NVP were more likely to be females than males, significantly younger and have a significantly higher baseline CD4 count than those on EFV as previously reported [8].

**Table 1:** Baseline characteristics of patients with and without a NNRTI-related skin rash.

Characteristic	NNRTI-related skin rash, n = 281	No NNRTI-related skin rash, n = 3,718	Total, n = 3,999	p-value
Age (years), median (IQR)	37 (31–45)	38 (32–45)	38 (32–45)	0.02
Gender (female), n (%)	218 (77.6)	2509 (68.2%)	2727 (68.2%)	0.0005
WHO clinical stage, n(%)				
1	11 (3.9)	261 (7.0)	272 (6.8)	0.36
2	33 (11.7)	450 (12.1)	483 (12.1)	
3	159 (56.6)	1980 (53.4)	2,139 (53.6)	
4	45 (16.0)	600 (16.2)	645 (16.2)	
no data	33 (11.7)	427 (11.3)	460 (11.5)	
Baseline BMI (kg/m^2^), median (IQR)	19.6 (17.4–22.3)	19.8 (17.5–22.7)	19.8(17.5–22.7)	0.36
Baseline CD4 count (cells/mm^3^), median (IQR)	102 (35–200)	135 (51–218)	132 (50–217)	0.001
CD4 count (cells/mm^3^), n (%)				
0–50	83 (29.5)	918 (24.7)	1,001 (25.0)	0.11
51–200	128 (45.6)	1647 (44.3)	1,775 (44.4)	
>200	69 (24.6)	1124 (30.2)	1,193 (29.8)	
no data	1 (0.3)	29 (0.8)	30 (0.8)	
Baseline serum creatinine, median (IQR)	79.6 (61.9–102.6)	85.7(69–106.1)	84.9(68.1–106.1)	0.03
Baseline serum ALT, median (IQR)	30 (20–43.5)	29 (20–43)	29 (20–43)	0.36
HBsAg serology, n (%)				
positive	33 (11.7)	263 (7.1)	296 (7.4)	0.0006*
negative	144 (51.2)	1708 (45.9)	1852 (46.3)	
not done	104 (37.1)	1747 (47.0)	1851 (46.3)	
NRTI backbone, n (%)				
ZDV + 3TC	132 (47.0)	1768 (47.6)	1900 (47.5)	0.69
D4T + 3TC	149 (53.0)	1941 (52.2)	2090 (52.3)	
Others^§^	0 (0.0)	9 (0.2)	9 (0.2)	
NNRTI, n (%)				
nevirapine	158 (56.2)	1463 (39.3)	1621 (40.5)	<0.0001
efavirenz	123 (43.8)	2255 (60.7)	2378 (59.5)	

NNRTI: Non-nucleoside reverse transcriptase inhibitor, NRTI: Nucleoside reverse transcriptase inhibitor, ALT: Alanine transaminase, AST: Aspartate transaminase, ZDV: zidovudine, 3TC: Lamivudine, d4T: stavudine. HBsAg: hepatitis B surface antigen. BMI: body mass index. § other NRTI backbones were TDF+3TC (n = 7), ddI+D4T (n = 1), ABC+3TC (n = 1).

### Incidence of skin rash

Of 341 patients experiencing a rash, 281 (82.4%) patients' rashes were deemed to be NNRTI-related. 264 patients had one episode and 17 had more than one episode of rash: 16 had 2 episodes and one patient had 3 episodes. The cumulative frequency of nevirapine-associated rash was 10.2% (n = 1,621) while efavirenz-associated rash was 5.6% (n = 2,378) with an overall frequency of 7.0% (n = 3,999) in the entire cohort. The overall incidence of NNRTI-related rash was 2.63 events per 100 person-years of follow up. The median time to onset of first episode of all skin rashes was 2 months (range 2-48 months). Reasons documented for 42 non NNRTI-associated skin rashes were HIV-associated nodular prurigo (n = 28), pruritic papular dermatitis (n = 7), varicella zoster (n = 3), co-trimoxazole-related rash (n = 2), tinea corporis (n = 1) and abacavir hypersensitivity (n = 1). The odds ratio of developing any skin rash on a nevirapine-based ART compared with efavirenz-based ART was 1.75 (1.40–2.19), p<0.0001.

### Severity of NNRTI-related skin rashes

Table 2 shows the frequencies of the various grades of NNRTI-related skin rash. 90 (30%) of the NNRTI skin rash were grade I, 70 (23%) were grade IIA, 122 (41%) were grade IIB, 15 (5%) were grade III and 2 (1%) were grade IV. Grade III skin rashes included Stevens Johnson syndrome (n = 7), generalised maculopapular rash with constitutional symptoms such as fever and malaise (n = 5), and maculopapular rash with hepatotoxicity (n = 3). The frequency of severe to life-threatening skin rash on nevirapine was 0.7% (n = 1,623) and on efavirenz was 0.2% (n = 2,376). There were no significant differences in the severity of NNRTI-related skin rash.

**Table 2:** Severity of NNRTI-related skin rashes among Ghanaian HIV patients.

Type	Nevirapine n = 165 (%)	Efavirenz n = 134 (%)	Total n = 299 (%)	Chi-square test p-value
**I**	44 (27%)	46 (34%)	90 (30%)	0.13
**IIA**	43 (26%)	27 (20%)	70 (23%)	0.27
**IIB**	66 (40%)	56 (42%)	122 (41%)	0.80
**III**	10 (6%)	5 (4%)	15 (5%)	0.35
**IV**	2 (1%)	0 (0%)	2 (1%)	0.20

Grade I –erythema/hyperpigmented rash localised, Grade IIA – diffuse maculopapular rash, Grade IIB- urticaria, Grade III- grade I,II + constitutional symptoms or angioedema or serum sickness-like reaction or Stevens Johnson Syndrome. Grade IV- toxic epidermal necrolysis.

### Risk factors for developing NNRTI-related skin rashes

**Figure 1:**
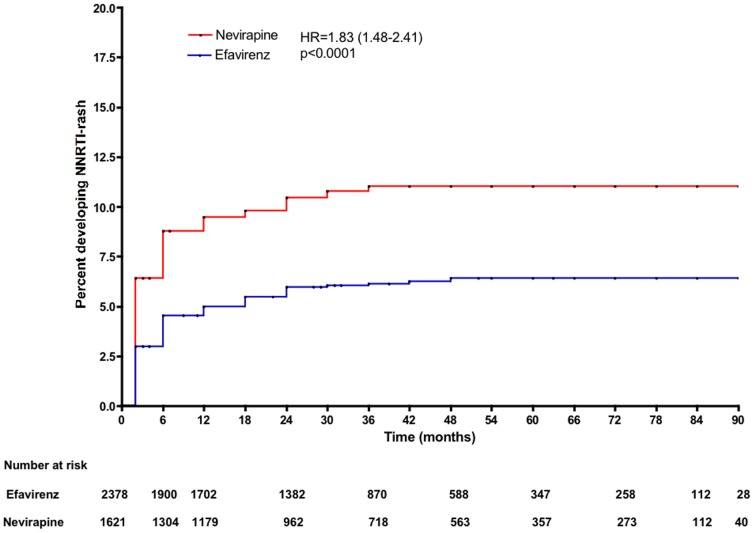
Kaplan-Meier estimates of risk for NNRTI-related skin rash according to NNRTI used.

**Figure 2:**
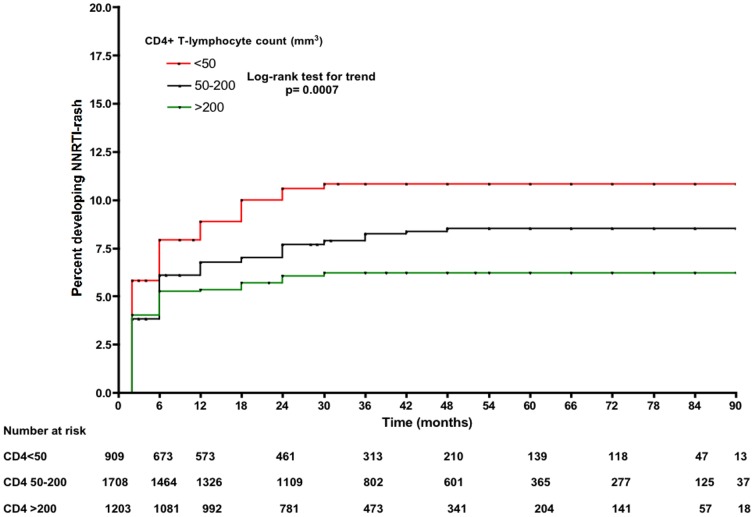
Kaplan-Meier estimates of risk for NNRTI-related skin rash according to CD4 count at baseline.

**Figure 3:**
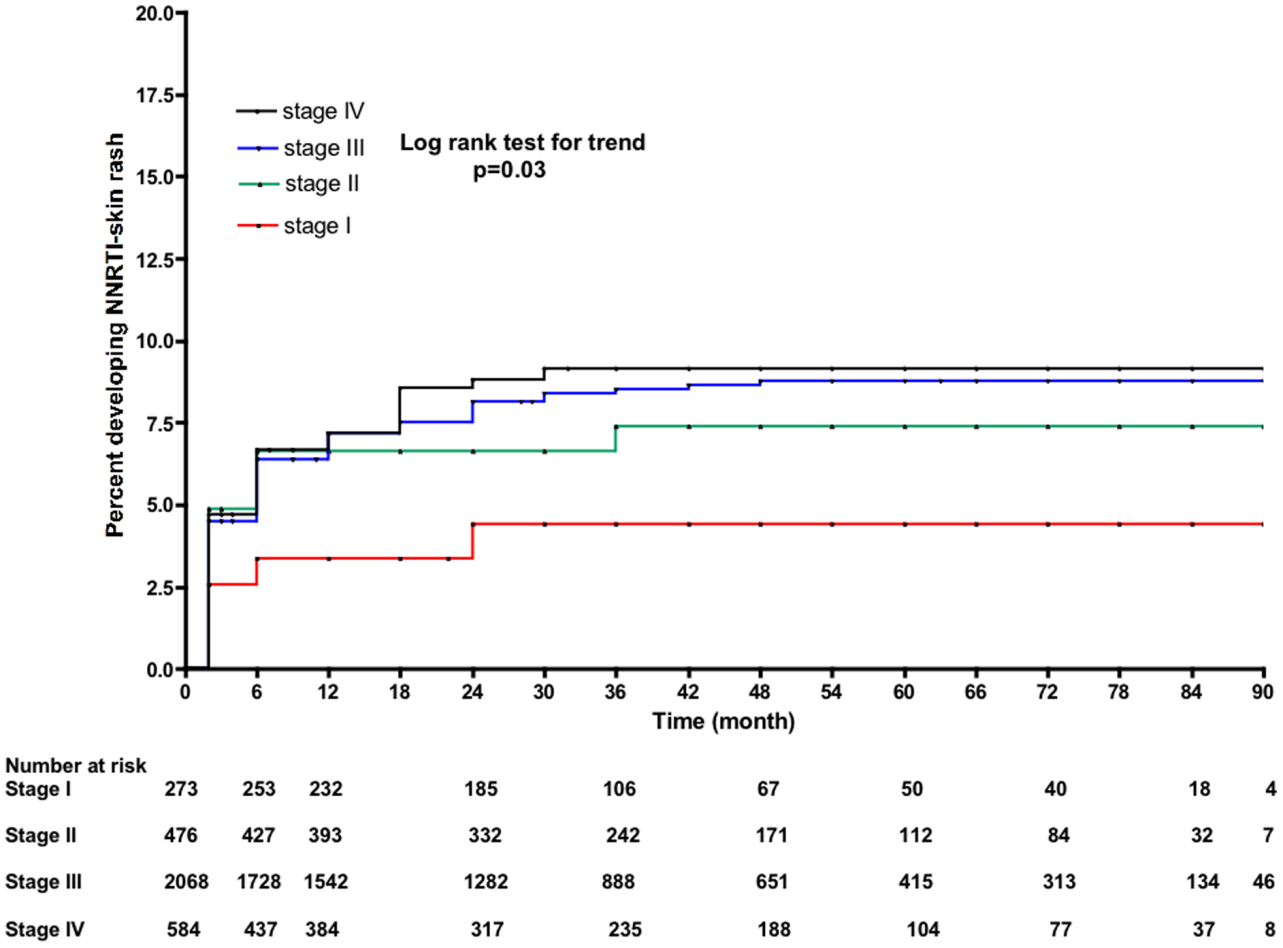
Kaplan-Meier estimates of risk for NNRTI-related skin rash according to WHO clinical stage at baseline.

In a multivariate model, use of NVP (adjusted HR 1.67, 95% CI 1.28–2.10), female gender (aHR 1.39, 95% CI 1.01–1.92), and CD4 counts (aHR 0.88, 95% CI 0.82–0.95, for each 50 cells/mm^3^ increment in baseline CD4) were associated with risk for developing a rash. Although HBsAg positivity was associated with NNRTI-related skin rash in the univariate model, this factor was not included in the final baseline multivariate model (n = 3,417) because the number of observations of this variable limited the power of the model (Table 3). In Kaplan Meier survival analyses, the use of nevirapine (rather than efavirenz), more advanced WHO stage and lower CD4 counts at baseline were all associated with an increased risk of developing a rash (Figures 1–3).

**Table 3:** Baseline risk factors associated with a NNRTI-related skin rash.

Variable	Unadjusted HR (95% CI)	p-value	Adjusted HR (95% CI)	p-value
**NNRTI**				
Nevirapine	1.83 (1.48–2.41)	<0.0001	1.67 (1.28–2.19)	0.0002
Efavirenz	1.00		1.00	
**Gender**				
Female	1.57 (1.18–1.97)	0.0013	1.39 (1.01–1.92)	0.04
Male	1.00		1.00	
**Age**				
<40 years	1.34 (1.06–1.71)	0.01	1.11 (0.85–1.45)	0.45
≥40 years	1.00		1.00	
**BMI per 5 kg/m^2^ increase**				
	0.86 (0.73–0.99)	0.05	0.94 (0.79–1.11)	0.47
**Clinical stage**				
(per each WHO stage higher)	1.21 (1.02–1.42)	0.03	1.13 (0.94–1.35)	0.20
**CD4 counts**				
(per 50 cells/mm^3^ increase)	0.89 (0.83–0.94)	0.0001	0.88 (0.82–0.95)	0.0005
**HBsAg status**				
positive	1.48 (1.03–2.49)	0.04	Not included*	-
negative	1.00			
**ALT per 10 IU/l higher**	1.01 (0.99–1.04)	0.33	-	-

* Not included as number of patients whose HBV sero-status was known was small.

### Characteristics of NVP versus EFV-associated rashes

Although risk factors associated with developing a NVP-associated rash have been well characterised, those for developing an EFV-associated rash still remain to be elucidated. Thus, an analysis was performed to identify whether risk factors predisposing to a NVP-associated rash were different from those of an EFV-associated rash. First, the median (range) time to the first reported rash was not significantly different for a NVP-associated rash, 2 months (2-36) compared with 2 months (2-48) for an EFV-associated rash, p = 0.12. Second, both NVP-associated and EFV-associated rashes occurred more often among patients starting ART with a low CD4 count, with the adjusted HRs (95% CI) of developing a NVP-associated or an EFV-associated rash with CD4 counts below 100 cell/mm^3^ of 1.48 (1.09–2.03) and 1.61 (1.09–2.39) respectively. Third, significant differences in associations were not found in the risk for either specific NNRTI-associated rash in relation to baseline age, NRTI backbone or ALT concentrations either at baseline or their changes at 2, 6 and 12 months on treatment (data not shown). Thus although NVP-associated rashes were more frequent than EFV-associated rashes, both appear to share common risk factors in this cohort.

### Immunological basis for NNRTI-related skin rash

A profound increase in CD4 counts during the initial phase of treatment has been proposed as one of the possible explanations for the development of NNRTI-related skin rash. Given our finding that the risk for developing skin rash was significantly higher for those with low CD4 counts at baseline, a comparison of the percentage and absolute changes in CD4 counts within the first 12 months of therapy was performed among those who developed NNRTI-skin rash and those who did not. The median (IQR) percentage change in CD4 counts at 6 months among patients who developed a NNRTI-related skin rash was +190% (93%–576%, n = 219) compared with +119% (57%–272.5%, n = 2,512), p<0.0001 for those without skin rash and +250% (103%–724%, n = 229) versus +143% (71% to 325.5%, n = 2,471) p<0.0001, respectively at 12 months. The median (IQR) of the absolute change in CD4 counts from baseline among patients who developed NNRTI-skin rash compared with those did not develop this event at 6 and 12 months were 172 (108–283) versus 159 (85–248) cells/mm^3^ p = 0.02, and 237 (148–336) versus 199 (114–304) cells/mm^3^, p = 0.0008 respectively. Thus compared with those who had no skin rash, patients who developed a NNRTI-related skin rash had more profound increases in CD4 counts from baseline.

### Treatment-limiting toxicity

Forty-four cases of NNRTI-related rash were treatment-limiting requiring modifications in therapy. These included 2 grade IV, 10 grade III, 16 grade IIB and 16 grade IIA skin rashes. These treatment-limiting adverse events were observed in 41 out of 165 (25%) patients on nevirapine compared with 3 out of 134 (2%) on efavirenz giving rise to a relative risk ratio (95% CI) of 11.10 (3.51–35.06), p<0.0001. Two patients (0.1% of those started on NVP) died from NVP-associated Stevens Johnsons' syndrome. There were 4 probable cases of cross-reactivity cutaneous reactions after substituting one NNRTI for the other (Table S1). All four cases involved a substitution of efavirenz on account of a nevirapine-associated rash. These rashes resolved initially upon discontinuing nevirapine but patients experienced another rash on efavirenz reported by clinicians as a NNRTI-related cutaneous hypersensitivity rash. However, in all cases these rashes resolved without discontinuation of efavirenz.

## Discussion

Treatment-associated toxicities have been identified as one of the major reasons for treatment discontinuation [4], [5], [9], [10] and an important predictor of poor adherence, which is particularly significant in resource-limited settings where second or third-line treatment options are often limited. The increased frequency of NNRTI-related skin rash in our study among patients on nevirapine, compared to those on efavirenz, is consistent with a number of other reports [4], [11], [12]. The reported frequencies of nevirapine-related rash ranged between 4%–54% of patients while that of efavirenz is between 4.6%–20% among different ethnicities [4], [11]–[20]. Thus the frequency of NNRTI-related skin rash within our cohort is at the lower end of the spectrum, along with that of several other studies in Africa [12]–[16]. Reasons for this lower than expected frequency of NNRTI-related skin rashes observed in this study could be under-reporting of adverse events being a retrospective study, loss-to-follow up (up to 24% in the present cohort), and deaths for which cause was unknown in some cases. Severe to life-threatening skin rash were reported at low frequencies (0.7% in nevirapine and 0.2% in efavirenz recipients) with 2 fatalities associated with nevirapine-related Stevens-Johnson syndrome.

The risk factors identified in association with skin rash on adjusted analysis included nevirapine use, female gender and low baseline CD4 counts. The latter association is in contrast with that reported among Thai and other patients [6], [18], [19], however consistent with a study in pregnant women in Cote d'Ivoire [16]. This difference could be due to differences in ethnicity and possibly genetic predispositions [21]. Female gender was significantly associated with NNRTI-related rash, as has been reported elsewhere [6], [15], [17], [22], [23] and it is thought to be related either to hormonal differences, gender related differences in cytochrome P450 metabolism and/or body size. However in many cohorts like ours, females are more adherent, less likely to withdraw from care and therefore may be more likely to report any adverse events, which may partially explain the observed increased risk.

One of the most important consequences of developing a hypersensitivity rash on NNRTI-based ART is the frequent need to stop the NNRTI, and the limitation of available antiretroviral drugs for second-line line therapy. Given the mild grade of rashes in most patients, particularly those on efavirenz, only a minority discontinued their NNRTI. There was however a striking difference in discontinuation rates between NNRTIs in that whilst very few patients developing a rash stopped efavirenz, a quarter stopped nevirapine (2.5% of all patients taking nevirapine), which was slightly lower than the rate observed in several other studies [17], [24]–[26]. The difference in discontinuation rates between the two NNRTIs was driven mainly by the low threshold for physicians to discontinue nevirapine since there were no significant differences in severity of rash on either NNRTI. Switching from nevirapine to efavirenz was associated with transient recurrence of rash in four patients but appeared to be generally safe.

A cell-mediated hypersensitivity reaction has been postulated as one of the possible mechanisms for the development of NNRTI related skin rashes, and a genetic predisposition has been proposed which may underlie this process [21]. Indeed in our cohort, a lower CD4 T-cell count at the time of initiating therapy followed by a more profound recovery in CD4 counts measured within 6 and 12 months were factors associated with a NNRTI-related skin rash. Thus our data support a possible mechanism akin to an immune reconstitution inflammatory syndrome (IRIS) where rapid restoration of CD4 T-cells elicit inflammatory responses to as yet to be identified antigenic epitopes, of which the NNRTI itself could be one among several other potential candidates.

In summary this study has shown that in Ghana, in contrast to the findings of other studies outside Africa, a low baseline CD4 count is risk factor for developing a NNRTI-related rash in patients starting ART. Furthermore, patients starting nevirapine are more likely to develop rashes and then more likely to discontinue therapy than those starting efavirenz. Given this and other treatment-limiting toxicities of nevirapine, superior clinical and virological outcome data for efavirenz and the reducing cost differential between these drugs in many resource poor countries, we believe efavirenz should replace nevirapine as the standard first-line NNRTI of choice in Ghana and probably also in other resource-limited countries as has been recently approved by the World Health Organisation [3].

## Supporting Information

Table S1 **Four cases of probable cross-reactive cutaneous reactions due to NNRTI class substitutions.**
(DOCX)
