# Supplementary material for: Incidence and Determinants of Nevirapine and Efavirenz-Related Skin Rashes in West Africans: Nevirapine's Epitaph?
Source: PLoS One. 2014 Apr 11;9(4):e94854. doi: 10.1371/journal.pone.0094854 (PMC3984248; doi:10.1371/journal.pone.0094854)
Supplement: Table S1 — Four cases of probable cross-reactive cutaneous reactions due to NNRTI class substitutions. (DOCX) [file pone.0094854.s001.docx]

**Table S1. Four cases of probable cross-reactive cutaneous reactions due to NNRTI class substitutions**

| **Gender** | **Age** | **WHO clinical**  **stage** | **CD4 count**  **/mm^3^** | **ART regimen** | **Time to 1^st^ rash (months)** | **Description and stage of 1^st^ rash** | **ART regimen changed to** | **Time to 2^nd^ rash**  **(months)** | **Description and stage of 2^nd^ rash** | **Outcome of 2^nd^ rash** |
| --- | --- | --- | --- | --- | --- | --- | --- | --- | --- | --- |
| Female | 39 | 4 | 26 | D4T+3TC+NVP | 2 | Diffuse pruritic rash, IIB | D4T+3TC+EFV | 6 | Hyperpigmented rash, I | Resolved without any intervention |
| Male | 40 | Not recorded | 210 | AZT+3TC+NVP | 2 | Generalised maculo-papular rash, IIA | AZT+3TC+EFV | 6 | Hyperpigmented, pruritic rash, I | Resolved without any intervention |
| Female | 52 | 3 | 7 | D4T+3TC+NVP | 2 | Generalised maculo-papular rash with mucosal involvement and ulceration, IV | D4T+3TC+EFV | 6 | Hyperpigmented rash, I | Resolved without any intervention |
| Female | 34 | 3 | 56 | AZT+3TC+NVP | 2 | Generalised maculopapular rash, IIA | AZT+3TC+EFV | 12 | Generalised pruritic rash, III | Resolved on antihistamines |
